# Chicoric acid ameliorates LPS-induced inflammatory injury in bovine lamellar keratinocytes by modulating the TLR4/MAPK/NF-κB signaling pathway

**DOI:** 10.1038/s41598-023-49169-z

**Published:** 2023-12-11

**Authors:** Xiang Lan, Dongdong Qi, Hao Ren, Tao Liu, Hong Shao, Jiantao Zhang

**Affiliations:** 1 College of Veterinary Medicine, Northeast Agricultural University, Harbin, China; 2 Heilongjiang Provincial Key Laboratory of Pathogenic Mechanism for Animal Disease and Comparative Medicine, Northeast Agricultural University, Harbin, China; 3 The Key Laboratory of Dairy Science of Education Ministry, Northeast Agricultural University, Harbin, China

**Keywords:** Pharmacology, Inflammation

## Abstract

Damage to lamellar keratinocytes, an essential cellular component of the epidermal layer of hoof tissue, can have a detrimental effect on hoof health and the overall production value of dairy cows. We isolated and cultured cow lamellar keratinocytes using the Dispase II and collagenase methods. We purified them by differential digestion and differential velocity adherent methods at each passaging and identified them by keratin 14 immunofluorescence. We established an in vitro model of inflammation in laminar keratinocytes using LPS and investigated whether chicoric acid protects against inflammatory responses by inhibiting the activation of the TLR4/MAPK/NF-κB signaling pathway. The results showed that cow lamellar keratinocytes were successfully isolated and cultured by Dispase II combined with the collagenase method. In the in vitro inflammation model established by LPS, the Chicoric acid decreased the concentration of inflammatory mediators (TNF-α, IL-1β, and IL-6), down-regulated the mRNA expression of TLR4 and MyD88 (P < 0.01), down-regulated the expression of TLR4, MyD88, p-ERK, p-p38, IKKβ, p-p65, p-p50 (P < 0.05), and increased the IκBα protein expression (P < 0.05). In conclusion, Chicoric acid successfully protected cow lamellar keratinocytes from LPS-induced inflammatory responses by modulating the TLR4/MAPK/NF-κB signaling pathway and downregulating inflammatory mediators.

## Introduction

Lameness is the most important issue in the global dairy industry^1^. Laminitis is the most common clinical hoof disease that triggers lameness and can lead to severe milk production loss^2^. The disease may be primary, but is more often secondary to rumen acidosis^3,4^. Additionally, it causes abnormal differentiation and inflammatory damage to the cells of the epidermis ^5^. Despite extensive research, the causative factors and pathophysiological mechanisms of bovine laminitis remain unclear. Therefore, there remains an urgent need for a standard in vitro model to further explore laminitis.

The dairy hoof lamellar tissue is a layer of connective tissue located between the hoof wall and the hoof bone, which is essentially a skin derivative and a unique structure composed mainly of skin dermal tissue and epidermal tissue extensions, creates a vast surface area with numerous cells tightly connected in the smallest volume^6^. The dermal lobules dependent on the distal phalanges (toes) are interdigitated with the keratinized lobules dependent on the medial aspect of the hoof wall to unite the hoof into a solid whole^7^. A bifurcated, interdigital interface between the epidermis and dermis suspends the bony phalanges from the keratinized hoof wall^8^.

Lamellar keratinocyte is an essential cellular component of the epidermal layer of hoof tissue, and the significant pathological change in experimentally induced laminitis is the apoptosis of keratinocytes in the epidermal layer of hoof tissue ^9^. In clinical laminitis in dairy cows, the cells of the epidermal layer are damaged by inflammation, and their ability to proliferate is reduced, resulting in a thinning and softening of the hoof shell, which causes other hoof diseases such as sole ulceration, hoof fissures, and white line fissures^10^. Currently, there have been studies on equine lamellar keratinocytes, Nicole^11^ used equine lamellar tissue explants to culture lamellar keratinocytes and analyzed the effect of lipopolysaccharide (LPS) on its supernatant lactate concentration, Carlos^12^ used collagenase to digest equine lamellar tissue. Despite the hoof's critical significance to cattle health, in vitro culture of dairy cow hoof lamellar keratinocytes has been given scant consideration.

Lipopolysaccharide (LPS) is a major component of the cell wall of Gram-negative bacteria and is effective in inducing inflammation^13^. Bacterial endotoxins are believed to contribute to the occurrence and development of laminitis ^14,15^. It has been demonstrated that after injecting bacterial endotoxin into cattle, the cuticle and epidermis showed lesion manifestations of acute laminitis ^16^. Elevated blood Lps concentrations in cows with hoof foliitis^5^. These findings suggest that endotoxin may play a role in the pathogenesis of laminitis in cattle. Nevertheless, the precise mechanism for this is not yet understood.

Chicoric acid (CA), scientifically known as dicaffeoyl tartaric acid, is a caffeic acid component isolated from dicotyledonous plants of the angiosperm family^17^, and has been shown to have various pharmacological effects such as anti-inflammatory^18,19^, antioxidant^20,21^, and immunomodulatory^22^. The clinical use of CA in dairy farming is rare, but experiments have demonstrated that the use of plants such as chicory and lettuce as feed can be effective in improving the efficacy and value of production in dairy cows^23,24^, but it has not been demonstrated whether this is related to the presence of CA in such plants. In addition, whether there is a positive effect of CA on bovine laminitis has not yet been demonstrated.

Of note is the high incidence of lameness in dairy cows and the greater adverse effects, which have a significant economic impact on dairy operations^25,26^. Not only is the dairy industry a major driver of the economy, but its products provide significant nutritional benefits to a growing population^27^. However, there are no effective methods for the prevention and treatment of hoof laminitis, and research on laminitis in dairy cows has focused mainly on the animal level, with little known about studies at the cellular level. For these reasons, we chose cow laminar keratinocytes as the research object, used LPS to establish an in vitro model of laminitis, and revealed its intrinsic mechanism as well as the mechanism of CA's protective effect on it.

## Result

### Morphological observations of laminar keratinocytes

**Figure 1:**
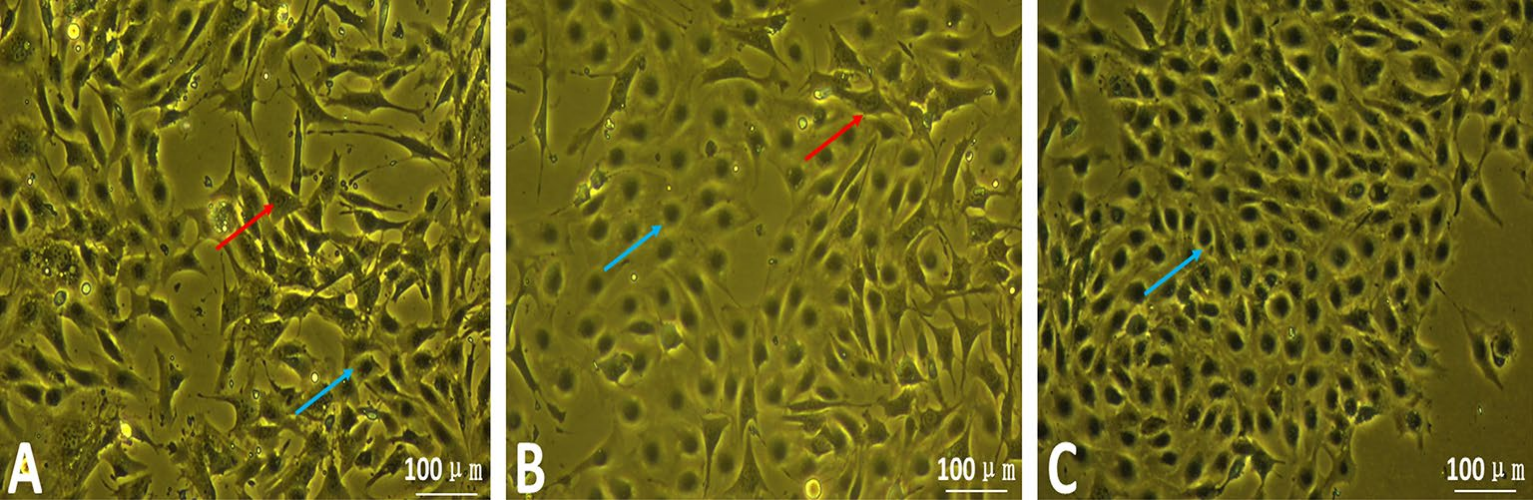
Fibroblasts are decreasing as they are passed on. (**A**): Primary Cells; (**B**): Passage 2 Cells; (**C**): Passage 4 Cells; → Fibroblasts; → Keratinocytes.

Fibroblasts were spindle-shaped or irregularly triangular with inconspicuous nuclei, while keratinocytes were flat and irregularly polygonal with translucent cytoplasm and clear round nuclei in the center of the cytoplasm. Cell morphology was observed after isolation using Dispase II combined with collagenase and purification using differential velocity adherent method and differential digestion method (Fig. 1), the proportion of fibroblasts was found to be decreasing and the proportion of keratinocytes to be increasing with cell passaging. By the fourth generation of cells, the percentage of keratinocytes had reached more than 90% and had formed the signature paving stone colonies of keratinocytes. The fibroblasts continued to decrease until they disappeared with continued purification.

### Immunofluorescence identification of keratin in laminar keratinocytes

**Figure 2:**
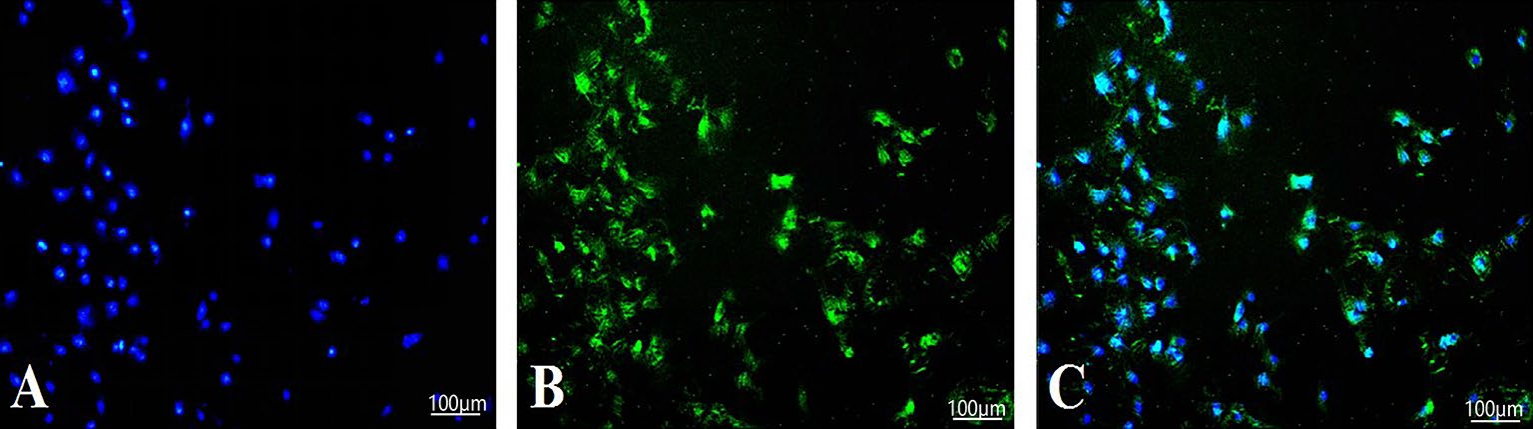
K14 Immunofluorescence Identification of keratinocytes (Passage 4 Cells) (**A**): DAPI; (**B**): K14; (**C**): Co-stained.

Fluorescence inverted microscopy showed that the majority of cells showed positive expression of green K14 (Fig. 2), indicating that the majority of cells were keratinocytes.

### Effects of LPS and CA on cell viability

**Figure 3:**
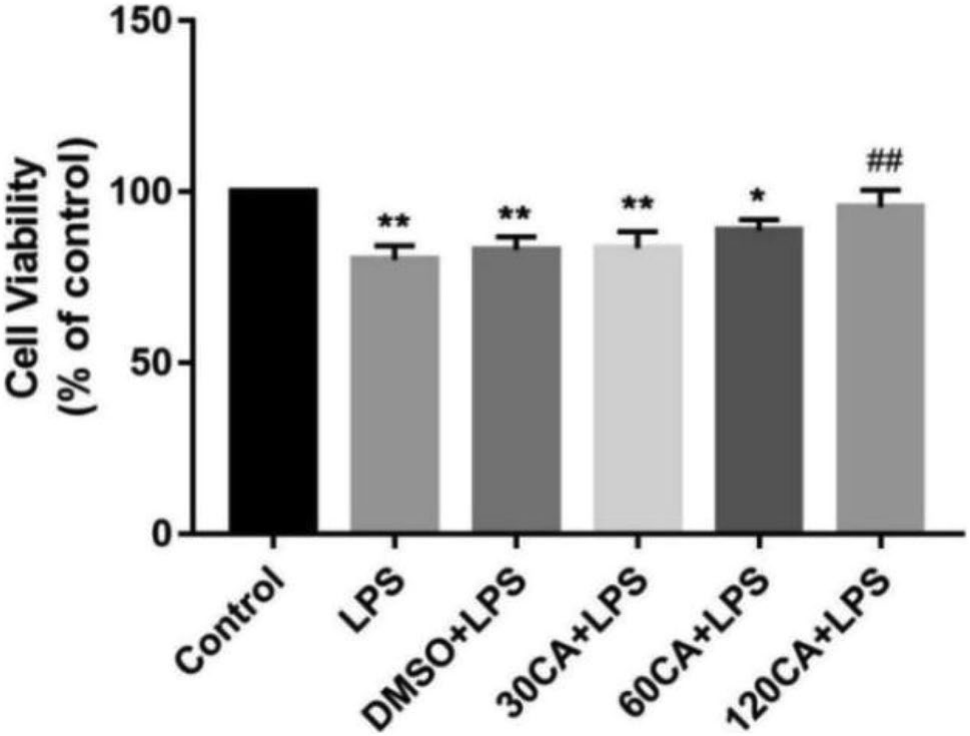
Effect of LPS and CA on cell viability. Notes: vs. Control, *P < 0.05, **P < 0.01; vs. LPS, ^##^P < 0.01.

Detection of cytotoxic effects by cck8. Cell viability was inhibited in all groups compared with the control group, but this inhibition was gradually restored with increasing CA concentration (Fig. 3). The results were shown to indicate that 10 μg/mL LPS treatment for 24 h induced a model of inflammation in lamellar keratinocytes, while CA restored the reduced cell viability.

### Effects of CA on LPS-induced expression of IL-1β, IL-6 and TNF-α in lamellar keratinocytes

**Figure 4:**
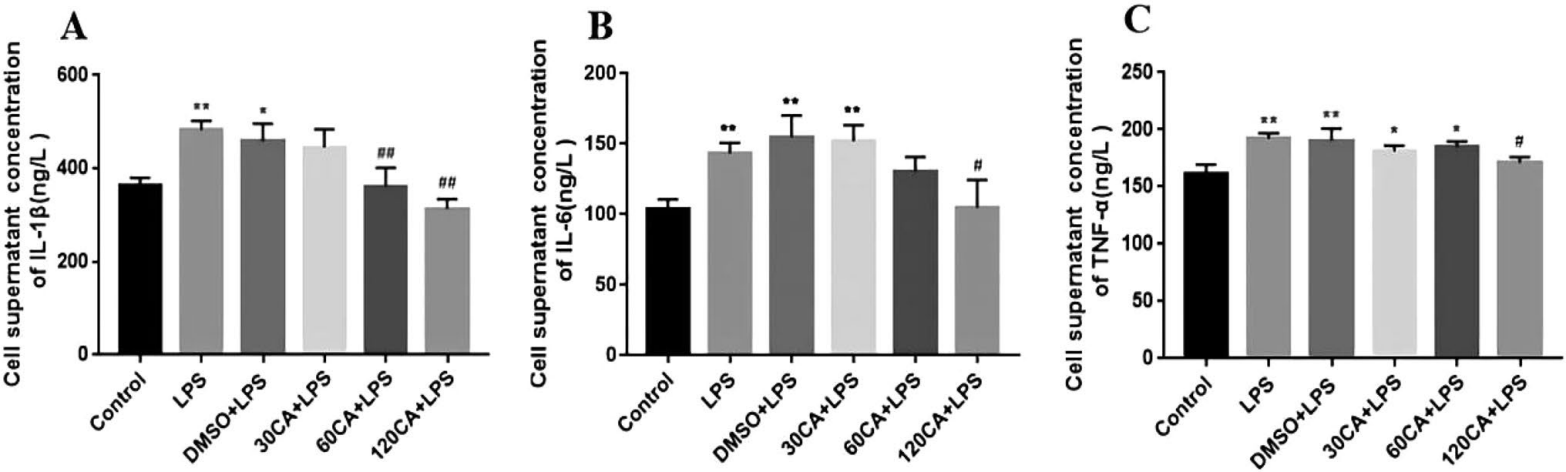
Cytokine levels (**A**) Levels of IL-1β (**B**) Levels of IL-6 (**C**) Levels of TNF-α. Notes: vs. Control, *P < 0.05, **P < 0.01; vs. LPS, ^#^P < 0.05; ^##^P < 0.01.

We measured the levels of the pro-inflammatory cytokines IL-1β, IL-6 and TNF-α by enzyme-linked immunosorbent assay. Figure 4 shows that LPS treatment significantly increased the levels of the three cytokines (P < 0.01 for IL-1β and IL-6, P < 0.05 for TNF-α) compared with the control group, suggesting that LPS induced an inflammatory response. In contrast, CA decreased the levels of these three cytokines. Compared with the LPS group, the 30 μg/mL CA + LPS group showed a significant decrease (P < 0.05) in IL-1β levels, but not in IL-6 and TNF-α levels. IL-1β and TNF-α levels were significantly decreased in the 60 μg/mL CA + LPS group compared with the LPS group (P < 0.05 and P < 0.01, respectively), but IL-6 levels were not decreased. The levels of all three cytokines were significantly decreased in the 120 μg/mL CA + LPS group compared with the control and LPS groups (IL-6 and TNF-α, P < 0.05; IL-1β, P < 0.01). These results suggest that CA reduced LPS-induced inflammatory cytokine production and that this reduction was dose-dependent.

### Effect of CA on TLR4 signaling pathway in LPS-induced inflammation in lamellar keratinocytes

**Figure 5:**
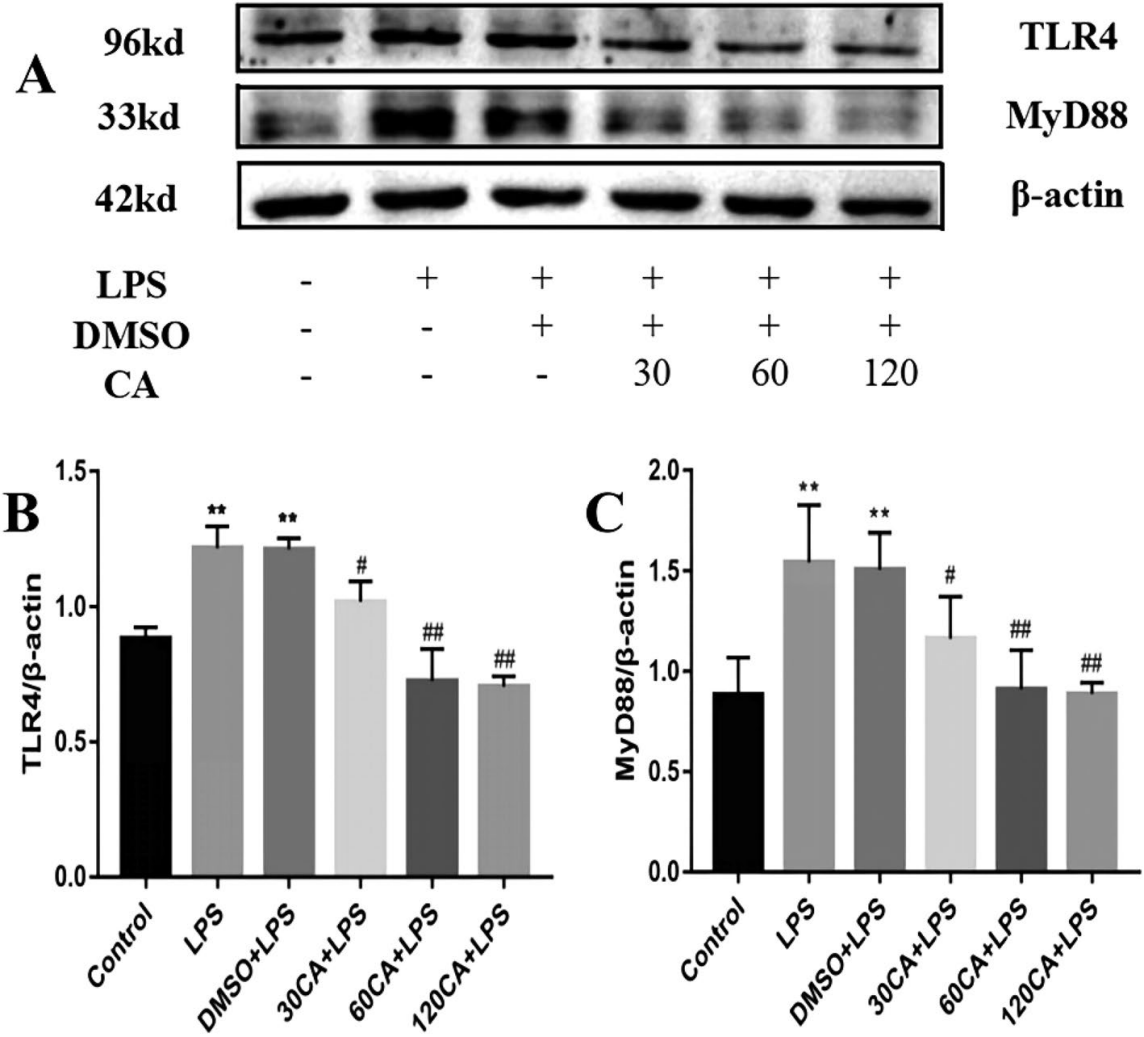
Relative protein levels of TLR4 signaling pathway (**A**) Western blotting to detect protein levels (**B**) TLR4 (**C**) MyD88.Notes: vs. Control, *P < 0.05; vs. LPS, ^#^P < 0.05; ^##^P < 0.01. (The full-length blots are presented in Supplementary file).

The TLR4 signaling pathway plays a key role in regulating the production of inflammatory cytokines. As shown in Fig. 5, TLR4 and MyD88 protein expression was significantly elevated in the LPS and solvent groups compared to the control group (P < 0.01). In contrast, the protein expression of TLR4 and MyD88 was significantly down-regulated in the 30 μg/mL CA + LPS group (P < 0.05), and significantly down-regulated in the 60 μg/mL CA + LPS and 120 μg/mL CA + LPS groups (P < 0.01). Shows concentration dependence.

### Effect of CA on MAPK signaling pathway in LPS-induced inflammation in lamellar keratinocytes

**Figure 6:**
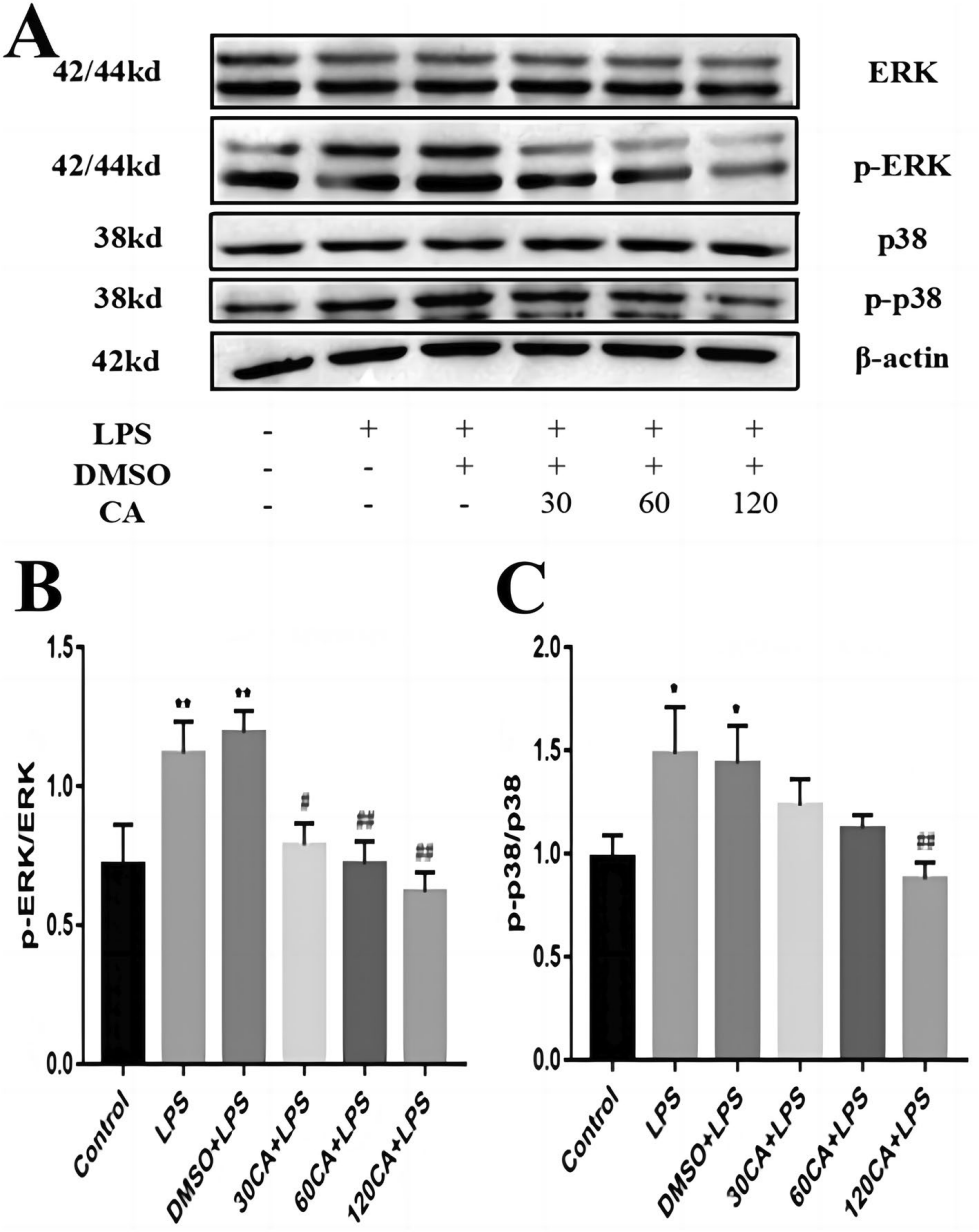
Relative protein levels of MAPK signaling pathway (**A**) Western blotting to detect protein levels (**B**) ERK, P-ERK.P-ERK/ERK (**C**) P-38, P-P38.P-P38/P-38. Notes: vs. Control, *P < 0.05; vs. LPS, ^#^P < 0.05; ^##^P < 0.01. (The full-length blots are presented in Supplementary file).

The protein expression of ERK and p38 was determined via Western blotting. Figure 6 shows that LPS treatment significantly increased the phosphorylation of ERK and p38 compared with control (P < 0.05), suggesting that LPS activated the MAPK pathway in lamellar keratinocytes. Conversely, CA reduced the phosphorylation of ERK and p38 in a dose-dependent manner. The expression of p-ERK and p-p38 in the CA + LPS group at 30 μg/mL was significantly reduced compared to that in the LPS group (P < 0.05). Moreover, the decrease was more significant in the CA + LPS group at 120 μg/mL (P < 0.01) (“Supplementary information”).

### Effect of CA on NF-κB signaling pathway in LPS-induced inflammation in lamellar keratinocytes

**Figure 7:**
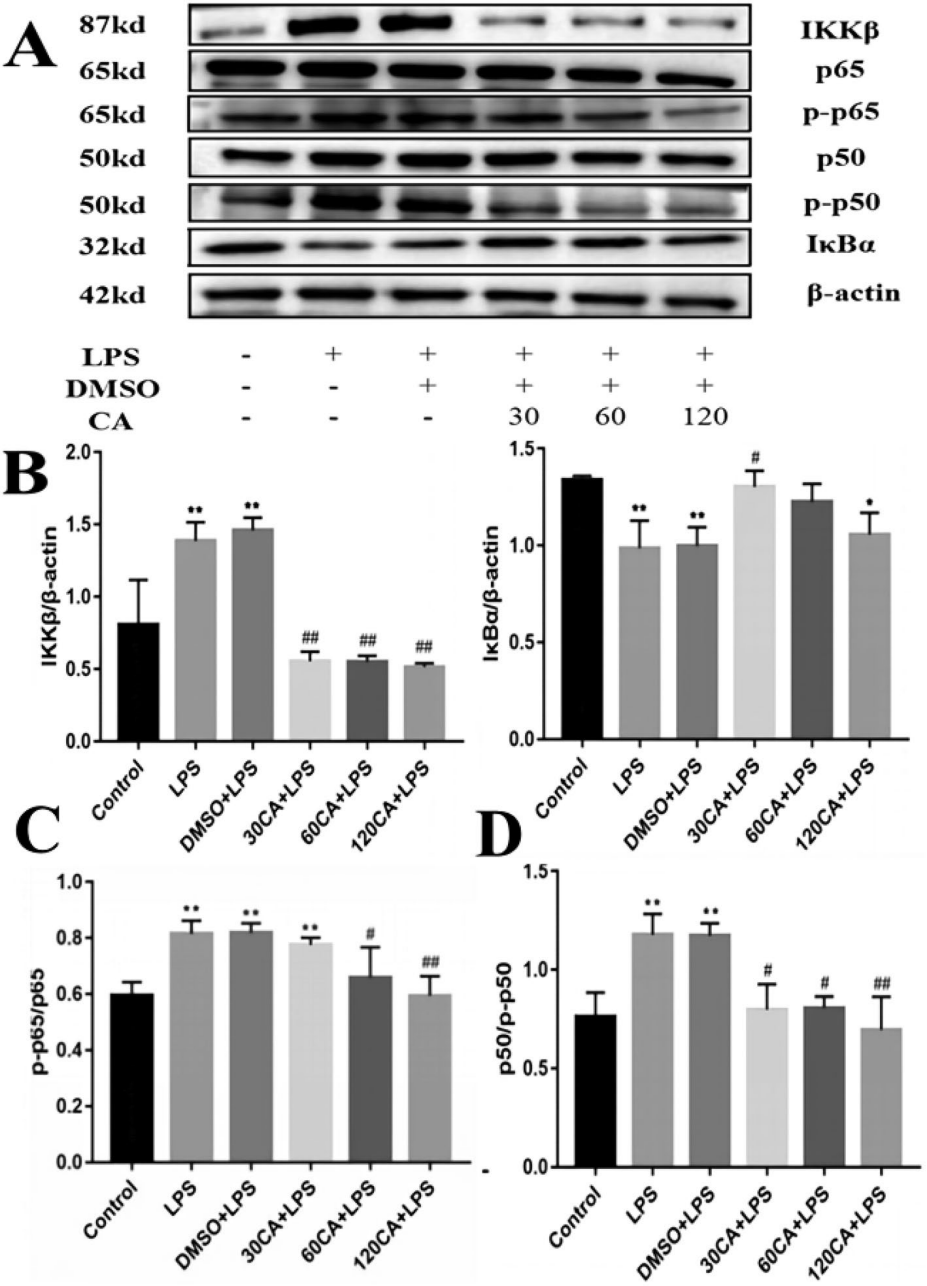
Relative protein levels of NF-κB signaling pathway (**A**) Western blotting to detect protein levels (**B**) IKKβ, IκBα (**C**) p65, p-p65, p-p65/p65 (**D**) p50, p-p50, p-p50/p50. Notes: vs. Control, *P < 0.05; vs. LPS, ^#^P < 0.05; ^##^P < 0.01. (The full-length blots are presented in Supplementary file).

We detected the protein expression of IKKβ, IκBα, p65 and p50, key components of the NF-κB pathway, in lamellar keratinocytes by Western blot. Figure 7 shows that LPS treatment significantly increased the expression of IKKβ and the phosphorylated forms of p65 and p50 and decreased the expression of IκBα compared with control (P < 0.01), indicating that LPS activated the NF-κB pathway in lamellar keratinocytes. In contrast, CA treatment decreased the expression of IKKβ and phosphorylated forms of p65 and p50 and increased the expression of IκBα in a dose-dependent manner. The expression of IKKβ, p-p65, and p-p50 was significantly decreased in the 30 μg/mL CA + LPS group compared with the LPS group (P < 0.05), whereas the expression of IκBα was significantly increased in the 30 μg/mL CA + LPS group compared with the LPS group (P < 0.05). There was a more significant decrease in the expression of IKKβ, p-p65 and p-p50 in the 60 μg/mL CA + LPS group compared to the LPS group (P < 0.01). The expression of IKKβ, IκBα, p-p65 and p-p50 recovered more significantly in the 120 μg/mL CA + LPS group compared with the LPS group (P < 0.01).

### Effect of CA on mRNA expressions of TLR4 and MyD88

**Figure 8:**
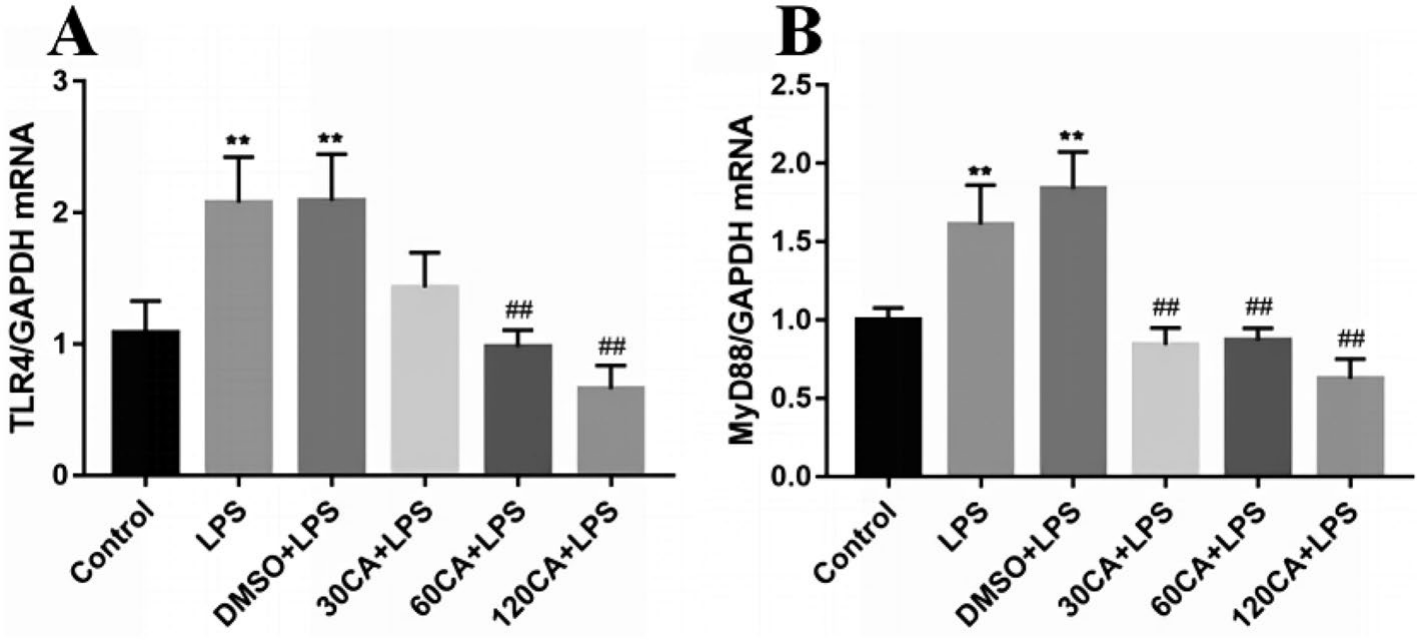
Gene expression of CA-treated TLR4 and MyD88 (**A**) TLR4 (**B**) MyD88. Notes: vs. Control, **P < 0.01; vs. LPS, ^##^*P* < 0.01.

As shown in Fig. 8, both the LPS and solvent groups highly significantly elevated the gene expression of lamellar keratinocytes TLR4 and MyD88 compared with the control group (P < 0.01); the 30 μg/mL CA + LPS group highly significantly down-regulated the gene expression of MyD88 compared with the LPS group (Fig. 8B, P < 0.01), and the 60 μg/mL CA + LPS group and the 120 μg/ mL CA + LPS group highly significantly down-regulated the gene expression of TLR4 and MyD88 (P < 0.01).

## Discuss

Type I collagenase is mainly used for the isolation of adipose, lung and epithelial tissues^28^. Laminar tissue is abundant in collagen fibers and has a complex collagen fiber support system with connecting and cushioning functions^29^, therefore, collagenase digestion is more suitable for the isolation of laminar tissue progenitor cells. Fibroblasts commonly appear in primary cultures, Visser and Pollitt ^30^ reported that splitting the epidermal layer from the dermal tissue in gathered samples whenever possible would decrease the occurrence of fibroblast contamination in keratinocyte cultures. Neutral protease, also known as Dispase, specifically hydrolyzes type IV collagen and fibronectin and separates the dermal-epidermal layer by digesting half-bridging granules, in skin tissue keratinocyte culture, neutral proteases can digest and separate the basal collagen layer, resulting in complete dermal-epidermal separation and thus purified keratinocytes^31^. Thus, the use of Dispase II in combination with collagenase removes collagen fibers, thereby reducing fibroblasts to a large extent and facilitating purification to obtain pure keratin-forming cells.

Fibroblasts are more sensitive to trypsin and can be shed from the cell culture flask in about 1 min, at which point discarding the digest can remove most of the fibroblasts ^32^, generally, keratinocytes are only susceptible to apposition after the cell vial is paved with rat tail collagen or a fibroblast trophoblast layer ^33^. Fibroblasts have a high ability to adhere to the wall and can adhere to the wall after an average of 1 h in a normal cell culture flask^34^. At this point, most of the fibroblasts can be removed by discarding the adherent cells and collecting the cells suspended in the culture medium. In this study, after several purifications, most of the cells obtained were keratin-forming cells.

Carter et al. demonstrated by quantitative proteomic analysis that K14/K5 is the predominant keratin expressed in horseshoe lamellar tissue^35^. Meanwhile, Yang et al. found that K14-positive cells in horseshoe lamellar tissue have typical progenitor cell characteristics and better differentiation ability, all of which suggest that K14 can be used as a specific marker for lamellar keratinocytes^6^. In the present study, most of the lamellar tissue cells passed to the fourth generation showed positive expression of K14, indicating that the resulting cells were keratinocytes.

It has been shown that levels of inflammatory factors such as TNF-α, IL-1β and IL-6 were elevated in the equine laminitis model induced by carbohydrate overload^36^ and black walnut overload^37^; furthermore, significant elevation of IL-6 and TNF-α has been reported in laminitis cows^5^, suggesting that cytokines play an important role in the pathologic development of laminitis. In this study, after LPS treatment, the levels of IL-1β, IL-6, and TNF-α in the cells were significantly increased, and cell viability was inhibited, which can be preliminarily concluded that the in vitro model of LPS-induced laminitis was established successfully.

LPS is an exogenous ligand for TLR4, and after LPS enters the organism, it first binds to the TLR4/MD-2 receptor to form a complex with high affinity, thus transducing the signal into the cell, which then activates the MyD88-dependent pathway and participates in the regulation of inflammatory response^38^, and it can also activate the MAPK and NF-κB signaling pathways to activate adaptive immunity ^39,40^. Yang et al. used LPS to stimulate endothelial cells and found that it could elevate TLR4 expression and activate the NF-κB signaling pathway^41^. MAPK signaling pathway is one of the important intersecting pathways of a variety of signaling pathways ^42^. Meanwhile, the activation of MAPK signaling pathway has an important connection with the pathogenesis of laminitis and the process of inflammatory response^43^. When the organism is subjected to external stimuli, various kinases activate IKKβ ^44^, which induces proteasomal phosphorylation and ubiquitination of IκBα, which induces the expression of IL-1β, IL-6, TNF-α and other pro-inflammatory factors expression ^45^. In the present study, key proteins of the TLR4-MAPK/NF-κB signaling pathway were altered after LPS treatment of laminar keratinocytes, LPS upregulated the mRNA levels of TLR4 and MyD88 in cow lamellar keratinocytes, which activated the downstream MAPK/NF-κB signaling pathway and caused inflammatory injury. In contrast, CA could inhibit the activation of the MAPK/NF-κB signaling pathway by down-regulating the expression of MyD88 in TLR4, which in turn inhibited the activation of the MAPK/NF-κB signaling pathway.

The primary method for treating laminitis currently involves administering antibiotic injections^46^, but misuse of antibiotics may lead to hazards, such as antibiotic residues in dairy products^47^. CA is a natural plant ingredient with anti-inflammatory and antioxidant properties. It can be studied for the prevention and treatment of laminitis in dairy cows. In a model of LPS-induced inflammation in yak peripheral blood mononuclear cells, Wang found that CA significantly reduced the secretion and mRNA expression of IL-1β, IL-6, and TNF-α^48^. It has been shown that pre-oral administration of CA to mice can inhibit the expression of TLR4 and MyD88, thereby inhibiting the activation of the NF-κB signaling pathway and decreasing the expression of inflammatory factors, thus achieving anti-inflammatory effects^49^. In addition, CA pretreatment inhibited IκBα degradation and NF-κB activation in macrophages^50^. CA significantly inhibited LPS-stimulated MAPK activation thereby suppressing inflammatory responses^51^. Our data again suggest that the anti-inflammatory effects of CA may act through the TLR4/MAPK/NF-κB signaling pathway. However, further research is still needed. In the present study, our data suggest that CA attenuates the inflammatory damage caused by LPS-induced lamellar keratinocytes and that this protection is concentration-dependent, the protective effect was enhanced with increasing CA concentration, with 120 μg/mL CA having the most pronounced protective effect. However, due to the small number of studies, relevant animal experiments are still needed to explore the optimal concentration of CA.

However, we conducted only in vitro experiments, which have limitations, and further in vivo studies on the therapeutic effects of CA in laminitis cows are required.

In summary, CA treatment significantly down-regulated the TLR4/MAPK/NF-κB signaling pathway, reduced the expression of inflammatory factors, and protected against LPS-induced inflammatory injury in keratinocytes. This experiment successfully established an in vitro model of laminitis in dairy cows, which can be further explored forresearch.

## Materials and methods

### Animal

The hoof tissues used in this study were obtained from 3 year old healthy Holstein cows (n = 4) without milking history at a slaughterhouse in Harbin, Heilongjiang Province, and the cows were inspected before slaughter to confirm that their hooves were healthy and intact. Slaughter sampling process in line with the "Livestock and Poultry Slaughter Management Regulations" and "Regulations on the Management of Laboratory Animals." All methods complied with good animal practices required by the Animal Ethics Procedures and Guidelines of the People’s Republic of China.

### Sample acquisition

**Figure 9:**
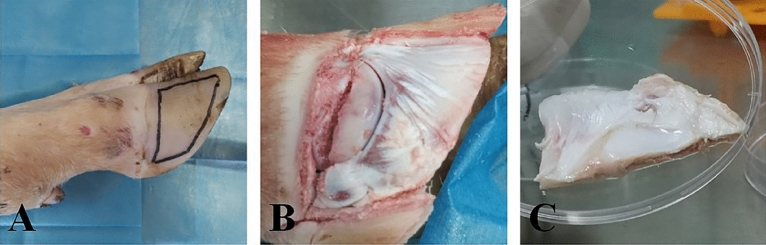
Sampling: (**A**) Cutting range of hoof wall; (**B**) Cut cow dairy hoof wall; (**C**) Removed dairy cow lamellar tissue.

Rinse the cow’s forelimbs with water and brush the hooves with a wire ball and test tube brush. Soak the cleaned forelimbs in a 10% chlorhexidine acetate solution for 10–15 min. Wipe the cow’s hoof with a 75% ethanol cotton ball. Saw the hoof wall and sole along the edge of the cow’s hoof wall with a saw blade soaked in 75% ethanol to form a trapezoidal opening (Fig. 9). Peel off the laminar tissue with a sterile surgical blade and rinse fragments and blood with PBS containing 5% penicillin. Place the removed laminar tissue in iodophor and 75% ethanol for 1 min each in turn. Immerse in PBS solution containing 5% penicillin–streptomycin and send to the laboratory.

### Keratinocyte isolation and culture

The laminar tissue was laid flat on the bottom of the Petri dish with the dermis on underneath, and the prepared Dispase II digestion solution was added until the laminar tissue floated and was placed at 4 °C overnight. The next day, the tissue was removed and placed on an ultra-clean table, the Dispase II was discarded, and the jellylike dermis was peeled off with ophthalmic forceps. An equal volume of collagenase digestion solution was added, and placed in a constant temperature water bath shaker at 37 °C for 2 h, and then the digestion was terminated by adding Dulbecco's Modified Eagle Medium (DMEM) complete medium.

The cell suspension was shaken well and poured into a 200-mesh copper mesh to filter the tissue, and the filtered liquid was poured into a centrifuge tube. It was centrifuged at 1500 rpm for 10 min, and the supernatant was aspirated and discarded. Add 3 mL of erythrocyte lysate, centrifuge again at 1500 rpm for 5 min, aspirate, and discard the supernatant. The cells were then inoculated with KSFM (Thermo Fisher Scientific, America) into T-25 cell culture flasks paved with rat tail collagen and incubated in a 37 °C, 5% CO_2_ cell culture incubator.

### Keratinocyte purification

Differential digestion method: When the cells were passaged, 1 mL of trypsin was added, digested for 1 min, then the digestion was terminated, rinsed three times, and l mL of trypsin was added again for digestion and observed under the microscope, and the digestion was terminated immediately after most of the cells were detached.

Differential velocity adherent method: The terminated digest was collected and centrifuged at 1500 r/min for 5 min, the supernatant was aspirated and discarded, the medium was added and resuspended by blowing, and the suspension was transferred into a new cell bottle and incubated in a 5% CO_2_ cell culture incubator at 37 °C for 1 h. After some cells were observed to be attached to the wall under the microscope, the unwalled cell suspension was aspirated in the bottle, centrifuged at 1500 r/min for 5 min, the supernatant was discarded, and KSFM complete culture medium was added. KSFM complete medium was added and the cells were inoculated.

### Keratinocyte identification

The cells were inoculated in 6-well plates. The cells were then fixed with 4% paraformaldehyde at room solution 3 times. They were treated with 0.5% Triton-X-100 (Beyotime Institute of Biotechnology, China) for 20 min at room temperature, aspirated 0.5% Triton-X-100, and rinsed 3 times slowly with PBS solution. Add 3% bovine serum albumin. for 30 min at room temperature. The cells were then incubated with K14 antibody (Affinity Biosciences, American) at 4 °C overnight. The next day, the primary antibody was aspirated and the cells were rinsed slowly 3 times with PBS solution. Cy3-labeled goat anti-rabbit IgG (1:500, Bimake, American) was added and incubated for 1 h at 37 °C, avoiding light. The secondary antibody was aspirated and the PBS solution was rinsed slowly 3 times. The nuclei were re-stained with 4’,6-diamidino-2-phenylindole (DAPI, Beyotime Institute of Biotechnology) under light-proof conditions, the DAPI was aspirated after 10 min, the PBS solution was rinsed slowly 3 times, and the slices were sealed with drops of anti-fluorescence quenching blocking solution (Beyotime Institute of Biotechnology, China), observed under a fluorescence microscope, and images were collected.

### Cell processing

After our initial research, In the LPS-induced cell damage study, lamellar keratinocytes were treated with 10 μg/mL of LPS (Beyotime Institute of Biotechnology, China) for 24 h. In protection experiments, cells were cultured in groups with KSFM medium containing 0, 30, 60, and 120 μg/mL of CA(Aladdin, American) and 6% DMSO (the solvent group that mimics the amount of DMSO contained in the maximum dose of CA) 6 h before the addition of LPS.

### Cell viability

Cell viability was assessed using the CCK-8 kit (Seven Biological, China) according to the manufacturer's instructions. First, cells were cultured in 96-well plates, then CCK-8 was added to the plates and incubated at 37 °C for 2 h. Finally, they were placed on an enzyme marker and the absorbance (OD) of each well was measured at 450 nm.

### Cellular Inflammatory Factor Content Assay

The levels of pro-inflammatory factors (IL-1β, IL-6, TNF-α) in the supernatant fluid of the cells in each group were detected by using ELISA kits (JINGMEI BIOTECHNOLOGY, China) according to the manufacturer's instructions, respectively.

### Western blot analysis

Total protein was obtained by radioimmunoprecipitaion assay (RIPA) cell lysis buffer, quantified by bicinchoninic acid (BCA) protein concentration assay kit (Beyotime Institute of Biotechnology, China), and 5 μL of the sample containing 5 μg of total protein was separated by SDS-PAGE and electrotransferred onto a nitrocellulose membrane. The membranes were closed with 5% skim milk for 1 h at room temperature and then incubated overnight at 4 °C with appropriate concentrations of protein-specific antibodies. After 6 washes with Tris-buffered saline plus Tween 20, the membranes were incubated with horseradish peroxidase-conjugated secondary antibody (Abcam, American) for 30 min at 37 °C and then washed again 6 times. The membrane was developed by placing the strips in a Tanon 5200 (Shanghai, China) exposer plate. The relative intensity of each protein strip/sample was quantified using Image J software. ERK, p-ERK, p38, p-p38, p65, p-p65, p50 and p-p50 purchased from Bimake Company, primary antibodies against TLR4, MyD88, IKKβ, IκBα and β-actin purchased from ABcam Company.

### Quantitative real-time PCR analysis

Laminar cells were inoculated into 6-well plates. After different treatments, total RNA was extracted with Trizol (Invitrogen, USA). The concentration and purity of total RNA (OD260/OD280 absorption ratio > 1.8) were assessed by a NanoDrop Ultra-Micro UV Spectrophotometer (Thermo Scientific, America). Subsequently, RNA was reverse transcribed to cDNA using a reverse transcription kit (NOVOPROTEIN, China) according to the manufacturer's protocol. Quantitative real-time PCR was performed using a 2 × NovoStart® SYBR Green Color qPCR SuperMix on a Roche Light Cycler 480 fluorescence quantitative PCR instrument (Roche, Basel Quantitative real-time PCR was performed using a 2 × NovoStart® SYBR Green Color qPCR SuperMix on a Roche Light Cycler 480 fluorescent quantitative PCR machine (Roche, Basel, Switzerland). The run program was set to 95 °C for 1 min, and then the following cycle was repeated 40 times: 95 °C for 20 s and 60 °C for 1 min. Gene expression was calculated using the 2-ΔΔCt method^18^. The mRNA expression of the target genes was standardized by GAPDH. Primerswere: TLR4, 5′-CTCTGCCTTCACTACAGAGA-3′ (forward), and 5′-CTGAGTCGTCTCCAGAAGAT-3′ (reverse); MyD88, 5′-TAGACAGCAGCATAACTCGGATAAA-3′ (forward), and 5′-GGTCAGACACGCACAACTTCA-3′ (reverse); GADPH, 5′-CACCTTCACCGTTCCAGTTT-3′ (forward), and 5′-GGTCATAAGTCCCTCCACGA-3′ (reverse).

### Data analysis

GraphPad Prism 7.0 software was used for data analysis and graphing. The experimental data results of each group were expressed as mean ± standard deviation (Mean ± SD), and ANOVA one-way and t-test were selected for significance of differences analysis. In the results, * and # represent P < 0.05, indicating significant differences, and ** and ## represent P < 0.01, indicating highly significant differences, all of which are statistically significant.

### Supplementary Information

Supplementary Information.

## Data Availability

The data presented in this study are available in this article.
