# Supplementary material for: Chicoric acid ameliorates LPS-induced inflammatory injury in bovine lamellar keratinocytes by modulating the TLR4/MAPK/NF-κB signaling pathway
Source: Sci Rep. 2023 Dec 11;13:21963. doi: 10.1038/s41598-023-49169-z (PMC10713547; doi:10.1038/s41598-023-49169-z)
Supplement: Supplementary file 1 — Supplementary Information. [file 41598_2023_49169_MOESM1_ESM.doc]

55kd

35kd

40KD

ERK 42/44kd

ERK FIG7


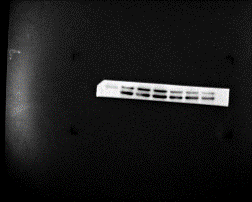


55

p-ERK 42/44kd

35

40KD

P-ERK FIG7


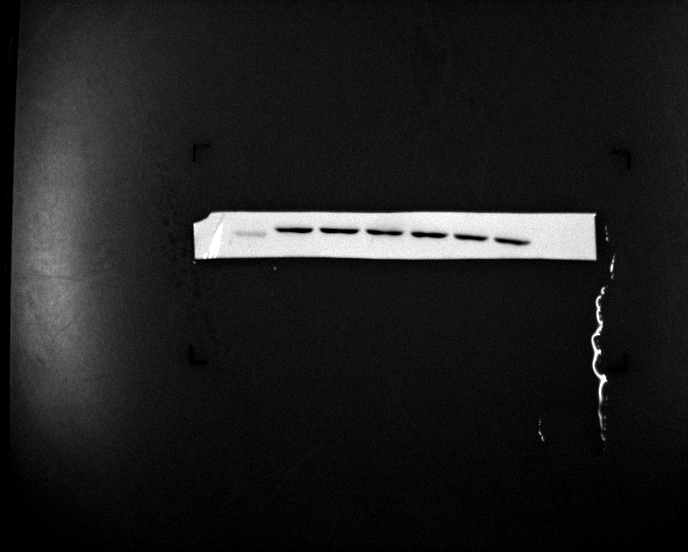


40kd

P38 38kd

25kd

35kd

P38 FIG7


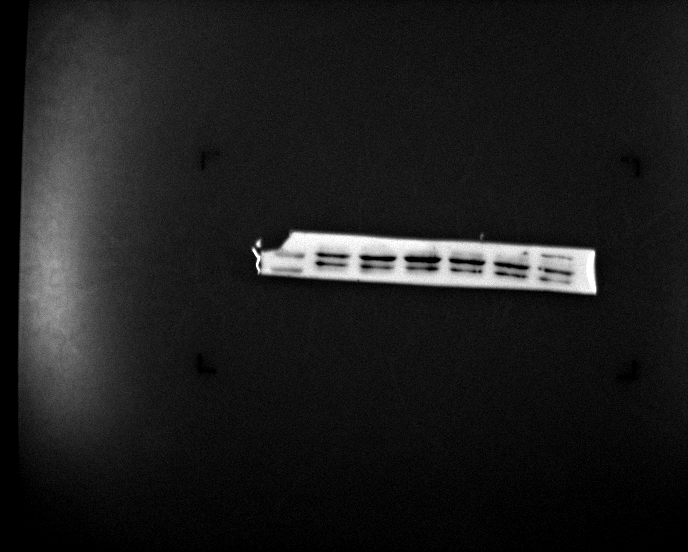


35kd

25kd

40kd

P38 38kd

24kd

P-P38 FIG7


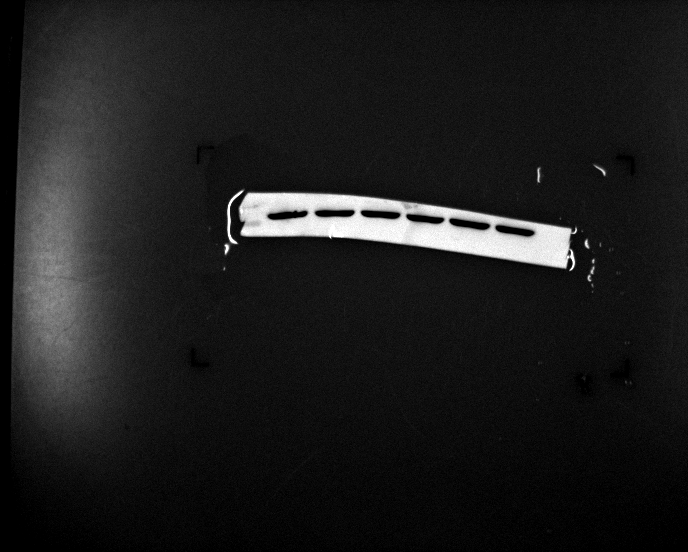


40kd

50-55kd

42kd β-actin

35kd

FIG7 β-actin


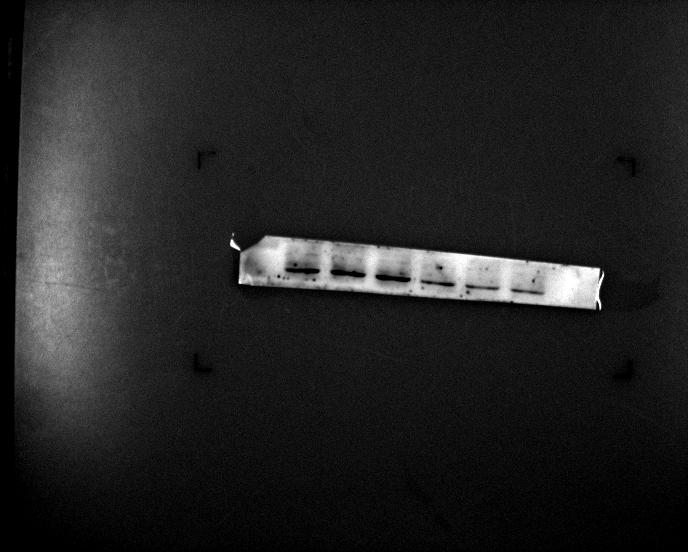


100KD

100KD

96KD TLR4

>70KD

TLRT FIG 6


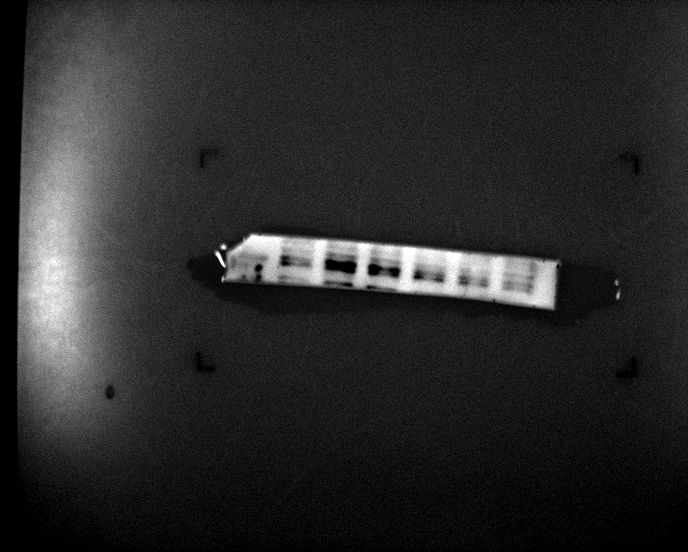


40KD

33KD MYD88

25

35KD

FIG6 myd88


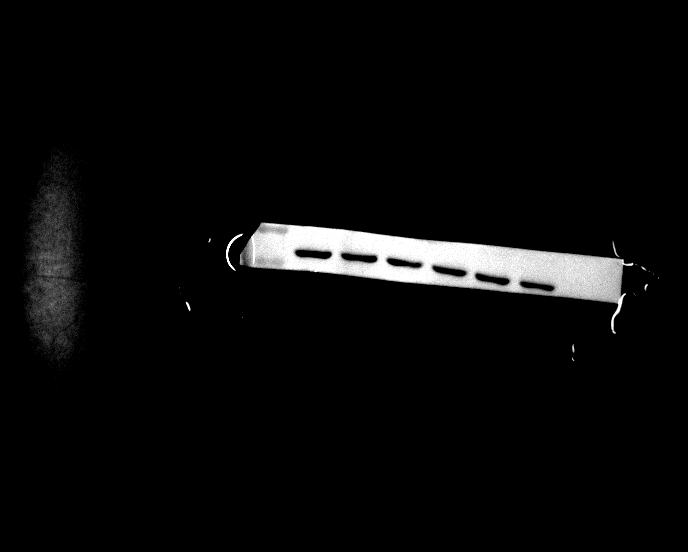


55KD

40KD

55KD

42KD β-actin

40KD

FIG 6 β-actin


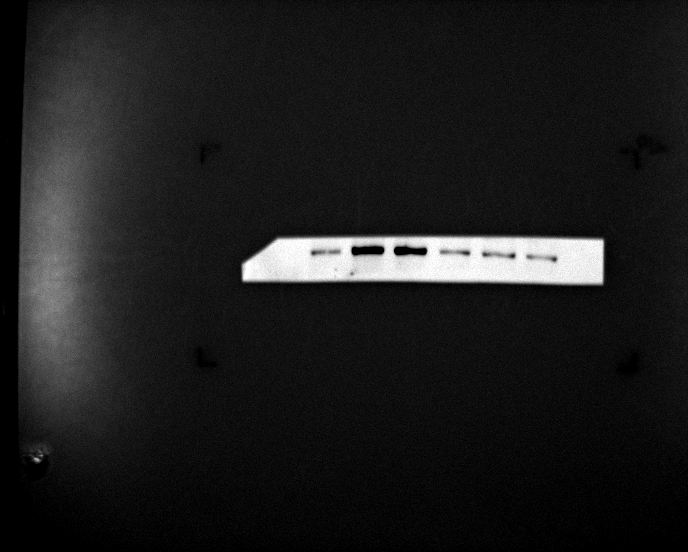


≈99KD

87KD IKKβ

≈75KD

Fig8 IKKβ


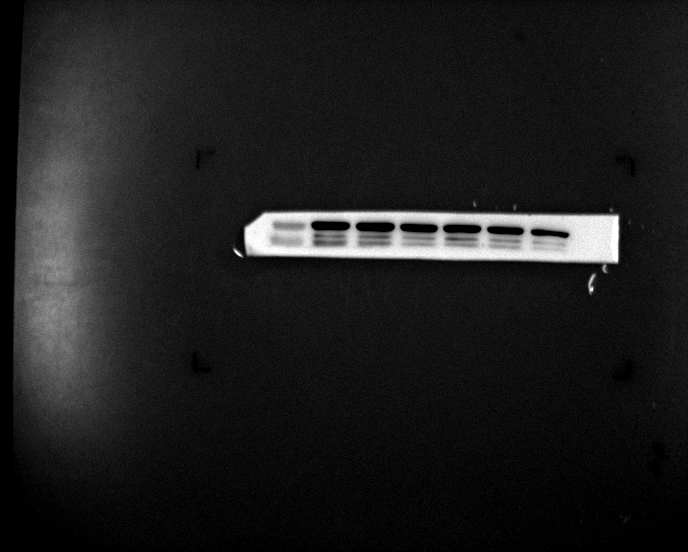


70kd

55kd

75kd

65kd p65

52kd

Fig8 p65


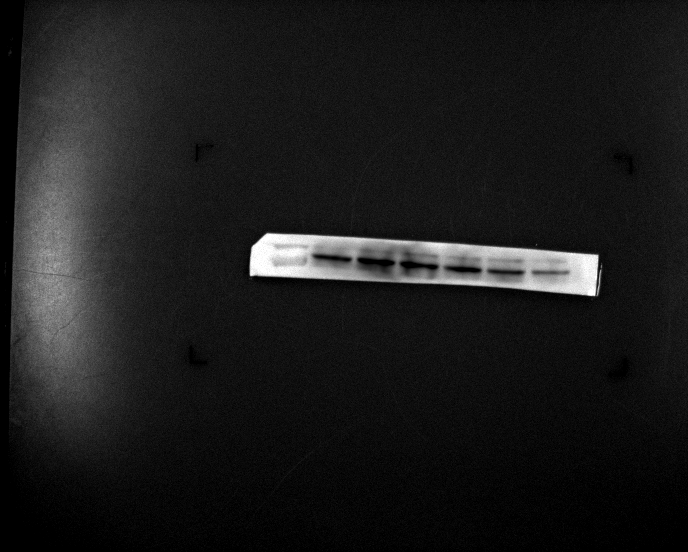


70kd

55kd

75kd

65kd p-p65

52kd

Fig8 pp65


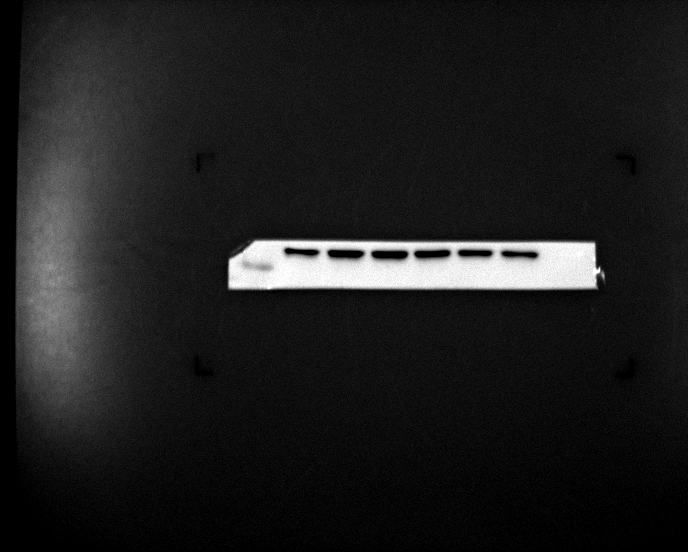


40kd

53kd

50kd p50

35kd

Fig8 p50


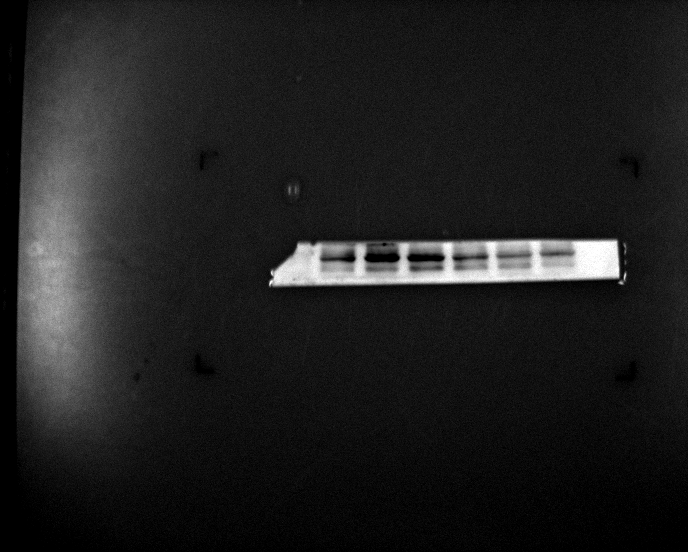


53kd

50kd p-p50

35kd

Fig8 pp50


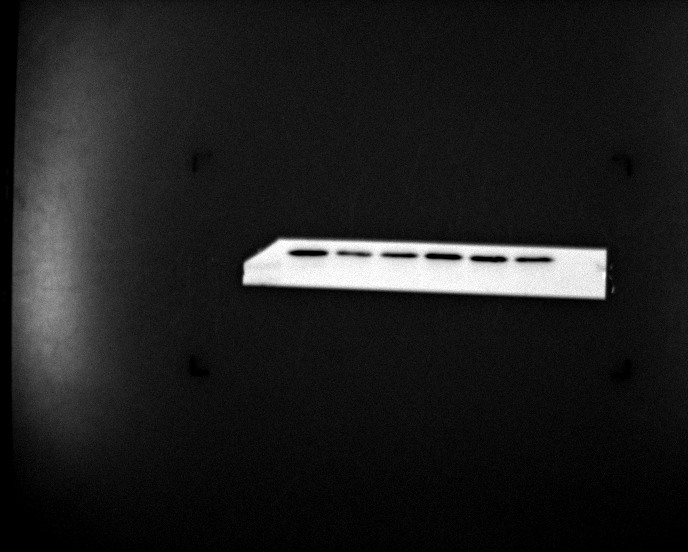


35kd

32KD ikbα

25kd

Fig8 ikbα


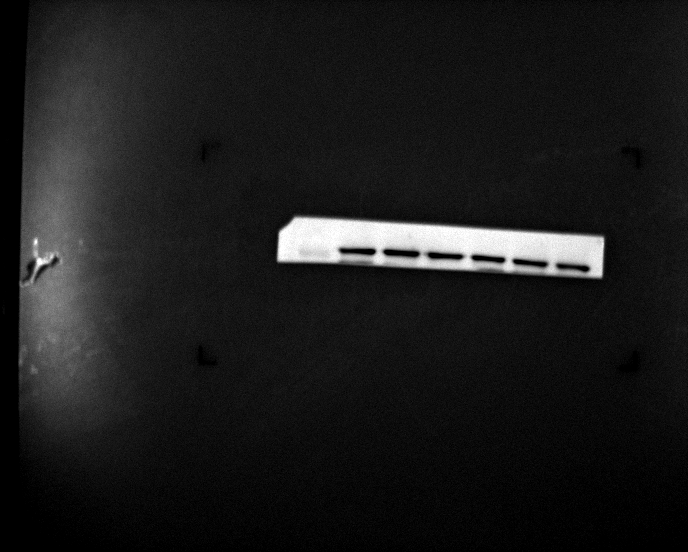


40kd

55kd

42KD ikbα

39kd

Fig8β-actin
